# Musicians Show Better Auditory and Tactile Identification of Emotions in Music

**DOI:** 10.3389/fpsyg.2019.01976

**Published:** 2019-08-28

**Authors:** Andréanne Sharp, Marie-Soleil Houde, Benoit-Antoine Bacon, François Champoux

**Affiliations:** ^1^École d’Orthophonie et d’Audiologie, Université de Montréal, Montreal, QC, Canada; ^2^Department of Psychology, Carleton University, Ottawa, ON, Canada

**Keywords:** emotion, music, auditory perception, tactile perception, brain plasticity

## Abstract

Musicians are better at processing sensory information and at integrating multisensory information in detection and discrimination tasks, but whether these enhanced abilities extend to more complex processes is still unknown. Emotional appeal is a crucial part of musical experience, but whether musicians can better identify emotions in music throughout different sensory modalities has yet to be determined. The goal of the present study was to investigate the auditory, tactile and audiotactile identification of emotions in musicians. Melodies expressing happiness, sadness, fear/threat, and peacefulness were played and participants had to rate each excerpt on a 10-point scale for each of the four emotions. Stimuli were presented through headphones and/or a glove with haptic audio exciters. The data suggest that musicians and control are comparable in the identification of the most basic (happiness and sadness) emotions. However, in the most difficult unisensory identification conditions (fear/threat and peacefulness), significant differences emerge between groups, suggesting that musical training enhances the identification of emotions, in both the auditory and tactile domains. These results support the hypothesis that musical training has an impact at all hierarchical levels of sensory and cognitive processing.

## Introduction

It is well established that musical training can lead to functional and structural changes in the brain, and that these changes correlate with improved music processing as measured by pitch, timing and timbre discriminations (for a review see Kraus and Chandrasekaran, 2010). Of particular importance to the present study, a number of studies have revealed that long-term musical training promotes brain plasticity and generates reorganization in regions related to audiotactile processing (e.g., Pantev et al., 2003; Baumann et al., 2007; Zimmerman and Lahav, 2012).

At the behavioral level, it has been shown that in detections tasks, musicians react faster to auditory and tactile stimuli (Landry and Champoux, 2017) and are also better at integrating auditory and tactile information (Landry et al., 2017). In auditory frequency discrimination tasks, musicians have lower threshold compared to controls (Spiegel and Watson, 1984), and this effect appears to be correlated with years of musical expertise (Kishon-Rabin et al., 2001).

To examine whether such discrimination enhancements extended to multisensory processing, Young et al. (2017) used a two-alternative forced choice task in which participants had to determine whether a pair of stimuli were the same or different. Participant could hear the stimuli, combined or not with a corresponding tactile stimulation transmitted through a glove. The results revealed that compared to controls, musician frequency discrimination threshold was improved significantly by the addition of tactile stimulation.

Recent results from our laboratory have confirmed such frequency discrimination enhancements in the auditory and audiotactile domains and have extended the latter by demonstrating that musicians were also better at discriminating tactile-only stimuli applied to the hand (Sharp et al., 2019). Taken together, these results suggest that musical training can have an impact on sensory processing, at least in detection or discrimination tasks. Whether such enhanced abilities can extend to more complex processes remains a matter of debate.

During the last decades, the study of emotions in music has become an increasingly popular research field. It is known that the ability to identify emotion in music starts early in life and that young children base their judgments on basic psychoacoustic cues such as tempo, loudness and pitch (Adachi et al., 2004). At 3 years of age, children are sensitive to the positive and negative connotations of music but their analysis is not yet sufficiently nuanced to distinguish between more specific emotions (Kastner and Crowder, 1990). It is only around 5 years of age that children begin to discriminate happiness and sadness (Terwogt and Van Grinsven, 1991).

Around 11 years of age, children are able to identify emotions at the adult level (Hunter et al., 2011). Since the identification of emotions in music is based on psychoacoustic cues and musical features, the possibility that musical training might enhance this ability has long been surmised. Indeed it appears that musicians are more accurate than non-musicians in the identification of emotions in music (Vieillard et al., 2008). Decline due to age in the identification of emotion in music is also less marked in musicians (Castro and Lima, 2014). Emotion identification abilities in musicians have not been examined further and the capacity of musicians to better identify emotion in music throughout different sensory modalities also remains to be determined.

The present study aims at investigating the auditory, tactile and audiotactile identification of various emotions in musicians using the stimuli of Vieillard et al. (2008) and tactile stimulation technology developed by Young et al. (2017). This study will be the first to examine tactile and auditory-tactile identification of emotion abilities in musicians versus controls.

## Methods

### Participants

Seventeen professional musicians (7 women, 10 men, average age = 28.9 years) and 17 matched non-musicians (8 women, 9 men, average age = 34.4 years) participated in the study. Non-musicians and musicians were matched for age, sex, handedness, educational level, and hearing thresholds. Only participants with less than 1 year of musical training were recruited for the non-musician (control) group. The sample size of this study is justified by the restrictive criteria used for inclusion in the musicians’ group. All musicians were working in the music field or studying music at the university level. The musicians specialized in piano (*n* = 9), guitar (*n* = 2), trumpet (*n* = 2), violin (*n* = 1), percussion (*n* = 1), flute (*n* = 1) and oboe (*n* = 1). They reported playing only one instrument (*n* = 4), playing two instruments (*n* = 2) or playing more than two instruments (*n* = 11). The average age of beginning to learn their first instrument was 7 years old. The average number of years of active practice of music was 20 years. Hearing thresholds were determined with an audiometer (Astera, GN Otometrics, Denmark). For both groups, pure-tone detection thresholds at octave frequencies ranging from 250 to 4000 kHz were within normal limits in both ears. The Research Committee for Health Sciences of the University of Montreal and the Center for Interdisciplinary Research in Rehabilitation of Greater Montreal approved all procedures, and each participant provided written informed consent. All experiments were performed in accordance with relevant guidelines and regulations.

### Stimuli and Procedure

The stimuli used in this study were developed by Vieillard et al. (2008). They are 56 melodies produced by a digital synthesizer in piano timbre. These instrumental stimuli were composed in the tonal musical tradition to express four emotions: happiness, sadness, fear/threat and peacefulness. The stimuli vary in mode, dissonance, pitch range, tone density, rhythmic regularity, and tempo but do not vary in performance-related expressive features (e.g., vibrato or variations of articulation/phrasing). Therefore the identification of emotions was based exclusively on the compositional structure. The mean duration of each stimuli was 12.4 s. All stimuli were originally validated by Vieillard et al. (2008) and were also cross-culturally validated by Fritz et al. (2009). These stimuli have been designed to elicit specific emotions that can be universally recognized.

The battery of Vieillard et al. (2008) was selected for this experiment because the four emotions evoked by the melodies are easily recognized and discriminated. Furthermore, all stimuli were validated cross-culturally by Fritz et al. (2009), and across age groups by Lima and Castro (2011). Finally, peacefulness is the most likely stimulus in this experiment to avoid a ceiling effect in musicians which show near perfection identification for better known emotions such has happy and sad.

Each of the 56 melodies were presented in a randomized order in three stimulation conditions: auditory-only, tactile-only and auditory-tactile. There were 14 stimuli for each type of emotion. For each stimuli, participants had to rate how much the melody expressed each of the four emotions on a 10-point intensity scale ranging from 0 (absent) to 9 (present). The four scales were presented immediately after each stimulus, and always in the same order (happy/sad/scary/peaceful). Each melody was presented only once in random order during each block (auditory only, tactile only and auditory-tactile) and no feedback was given. All conditions for stimulation and emotion were randomized. For example, one participant started in the tactile condition with a peaceful stimulus, while another started in the auditory condition with a sad stimulus. To exactly replicate the standardized task of Vieillard et al. (2008), the order of the scale presented after each stimulus was not counterbalanced.

Participants were seated in a soundproof room and stimuli were presented via headphones (TDH-39, Diatec, Canada) for the auditory-only condition, via a vibrating glove device for the tactile-only condition, and via both headphones and a vibrating glove for the auditory-tactile condition. During the tactile-only condition, white noise was presented via headphones and the participant wore earplugs. The participant had to adjust the volume during practice trials so as not to hear the vibrating glove.

The vibrating glove was a replication of the glove used by Young et al. (2017) and was equipped with six independent audio-haptic voice-coil exciters. The voice-coil transducers (TEAX14C02-8 Compact Audio Exciter) had a diameter of 14 mm and were designed to deliver vibrotactile output. The frequency range of these speakers is 300 to 20,000 Hz. Stimuli were sent via a Dayton Audio DTA3116S Class D Micro Mini Amplifier (2 × 15 W), linked via an audio cable to the software Psyscope 1.2.5 (Cohen et al., 1993) on a Mac computer.

### Analysis

The percentage of accurate responses, defined as the highest rating score for a melody corresponding to the intended emotion, was calculated for each participant for each emotion. For example, given a happy melody and a rating of Happy = 7, Sad = 3, Fear = 2, Peaceful = 6, the response would be counted as correct, whereas Happy = 6, Sad = 3, Fear = 2, Peaceful = 7 would be counted as incorrect. The same rating could never be used twice for any of the melody ratings.

An ANOVA was used as an omnibus test to compare the percentage of accurate responses for stimulation conditions and emotions as within-subject factors and groups as a between-subject factor. A multivariate analysis of variance was used to compare the percentage of accurate responses between groups. To provide an estimation of multisensory benefits compared to unimodal stimulation, the increase in performance was measured by subtracting the score in the auditory only condition from the score in the auditory-tactile condition. The results provide an estimation of the contribution of tactile stimulation.

## Results

**FIGURE 1:**
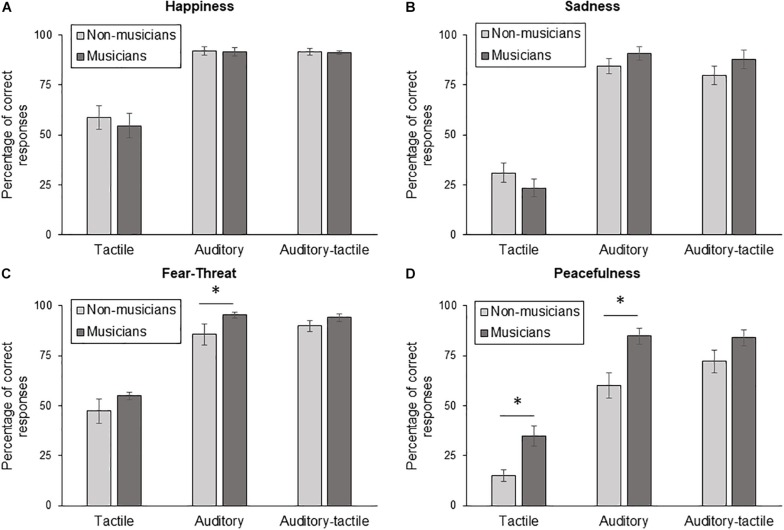
Percentage of correct responses for non-musicians and musicians for the three test conditions(Tactile, Auditory, Auditory-tactile) for **(A)** happy, **(B)** sad, **(C)** fear/threat, and **(D)** peacefulness. Error bars represent the mean standard error. ^∗^*p* < 0.05.

Figure 1 displays the percentage of accurate responses for auditory, tactile and auditory-tactile conditions for each of the emotions. An ANOVA for stimulation conditions, emotions and groups was used as an omnibus test. There was a significant difference between groups (*F*(1,32) = 10.834, *p* = 0.002). There was also a significant interaction between the condition and emotion variables (*p* < 0.0001).

The multivariate analysis of variance used to compare the percentage of accurate responses revealed a statistically significant difference in conditions based on Group (*F*(12, 21) = 2.585, *p* = 0.027; Wilk’s Λ = 0.404, partial η^2^ = 0.596). Table 1 shows that there were significant differences between groups for fear/threat auditory, peacefulness auditory and peacefulness tactile whereas no significant differences between groups were found in the other conditions.

**TABLE 1:** Statistical results from the multivariate analysis of variance used to compare percentage of accurate responses between groups (auditory, tactile, auditory-tactile) for all four emotions.

	**Auditory**	**Tactile**	**Auditory-Tactile**
Happy	*F*(1, 32) = 0.313	*F*(1, 32) = 0.023	*F*(1, 32) = 0.020
	*p* = 0.579	*p* = 0.881	*p* = 0.887
	partial η^2^ = 0.0.010	partial η^2^ = 0.0.001	partial η^2^ = 0.0.001
Sad	*F*(1, 32) = 3.010	*F*(1, 32) = 1.859	*F*(1, 32) = 0.797
	*p* = 0.092	*p* = 0.182	*p* = 0.379
	partial η^2^ = 0.0.086	partial η^2^ = 0.0.055	partial η^2^ = 0.0.024
Fear/Threat	*F*(1, 32) = 4.23	*F*(1, 32) = 2.379	*F*(1, 32) = 1.940
	***p* = 0.048^∗^**	*p* = 0.133	*p* = 0.173
	partial η^2^ = 0.117	partial η^2^ = 0.0.069	partial η^2^ = 0.0.057
Peacefulness	*F*(1, 32) = 15.838	*F*(1, 32) = 8.432	*F*(1, 32) = 1.531
	***p* < 0.001^∗^**	***p* = 0.007^∗^**	*p* = 0.225
	partial η^2^ = 0.0.331	partial η^2^ = 0.209	partial η^2^ = 0.0.046

*Bold and ^∗^*p* < 0.05.*

Uncorrected *t*-tests revealed that for both groups, mean percentage of responses was above chance for auditory and auditory-tactile stimulation conditions for all type of emotions (*p* < 0.001). For tactile stimulation, uncorrected *t*-tests revealed that the mean percentage of responses was above chance for both groups for happy (*p* < 0.001) and fear/threat emotions (controls: *p* = 0.002, musicians: *p* < 0.001), but not for sad (controls: *p* = 0.153, musicians: *p* = 0.747). Finally, an uncorrected *t*-test showed that musicians were performing above chance for tactile stimulation for peaceful emotion (*t*(16) = 2.170, *p* = 0.045) while on the contrary, another uncorrected *t*-test showed that controls were performing below chance for tactile stimulation for peaceful (*t*(16) = −4,629, *p* < 0.001).

For the happiness and sadness conditions, no increases in performance were observed in the auditory-tactile compared to the auditory-only condition in either groups (mean under 0%). For sadness and peacefulness, there were increases measured for controls (Sadness: 4% and Peacefulness: 12%), but not for musicians (mean under 0%). After correcting for multiple comparisons he increase in performance between auditory and auditory-tactile stimulation was not significant for either musicians or controls (see Table 2 for more details).

**TABLE 2:** Mean percentage of increase in performance from adding tactile stimulation to auditory stimulation (auditory-tactile performance – auditory only performance).

	**Group**	**Mean (%)**	**Standard error of the mean**	***t*-Test: Auditory versus Auditory-tactile performance**
Happiness	Controls	–0.50	2.04	*t*(16) = 0.833, *p* = 0.417
	Musicians	–0.39	2.15	*t*(16) = 0.190, *p* = 0.851
Sadness	Controls	–4.59	5.96	*t*(16) = 2.048, *p* = 0.057
	Musicians	–2.87	3.00	*t*(16) = 0.975, *p* = 0.344
Fear/Threat	Controls	4.24	3.51	*t*(16) = −1.357, *p* = 0.193
	Musicians	–1.20	1.52	*t*(16) = 0.824, *p* = 0.422
Peacefulness	Controls	12.19	5.73	*t*(16) = 2.318, *p* = 0.034
	Musicians	0.78	3.50	*t*(16) = 0.235, *p* = 0.818

ANOVAs were used as an omnibus test to compare the number of errors between groups for each expected emotion (4). The dependent variable was the number of errors and independent variables were groups, stimulation conditions and categories of the emotion scale. The ANOVAs for happiness (*F*(1,32) = 0.141, *p* = 0.710), sadness (*F*(1,32) = 0.196, *p* = 0.661) and fear/threat (*F*(1,31) = 3.061, *p* = 0.090) revealed no differences between groups. There was a significant difference between groups for peacefulness (*F*(1,32) = 10.691, *p* = 0.003). *t*-Test analysis revealed differences between groups for sadness when the expected emotion was peacefulness. This emotion had the higher rate of error for both groups. The difference between groups was the number of error, but not the type of emotion wrongly associated with peacefulness. In all conditions, both group were doing the same kind of errors for each type of emotion as shown in Table 3. In the auditory stimulation condition, for both groups, the emotion with which happiness and sadness was most often confused with was peacefulness. Similarly, for both groups, the emotion with which fear/threat and peacefulness were most often confused with was sadness. Results were the exact same in the auditory-tactile stimulation. In the tactile stimulation condition, for both groups, the emotion with which happiness was most often confused with was fear/threat. For all other emotions in the tactile stimulation condition, errors were distributed across the other three type of emotions. The missing values in Table 3 are due to the fact that it was not possible to categorize some errors, because some participants were giving a 0 score to all types of emotions in the scale for a few trials.

**TABLE 3:** Mean percentage of correct responses and mean percentage of errors per emotion classified by the type of emotion wrongly identified.

	**Auditory**							
	**Happiness**				**Sadness**			
	**Correct**	**Sadness**	**Fear/Threat**	**Peacefulness**	**Correct**	**Happiness**	**Fear/Threat**	**Peacefulness**
Controls	92.02	0.84	0.84	6.72	86.98	0.42	2.10	7.14
Musicians	91.61	0.84	0.00	7.56	90.76	0.42	2.10	7.14

	**Fear/Threat**				**Peacefulness**			
	**Correct**	**Happiness**	**Sadness**	**Peacefulness**	**Correct**	**Happiness**	**Sadness**	**Fear/Threat**

Controls	84.45	7.14	10.50	1.68	61.77	19.75	**23.93^∗^**	0.84
Musicians	95.38	0.00	3.36	0.42	84.87	6.30	**8.86^∗^**	0.00

	**Auditory-tactile**							
	**Happiness**				**Sadness**			
	**Correct**	**Sadness**	**Fear/Threat**	**Peacefulness**	**Correct**	**Happiness**	**Fear/Threat**	**Peacefulness**

Controls	90.23	0.00	0.00	3.78	76.47	0.42	2.94	10.50
Musicians	91.19	1.26	0.00	6.30	87.84	0.00	4.62	5.46

	**Fear/Threat**				**Peacefulness**			
	**Correct**	**Happiness**	**Sadness**	**Peacefulness**		**Happiness**	**Sadness**	**Fear/Threat**

Controls	89.50	0.42	3.36	0.84	75.64	9.24	17.65	0.84
Musicians	94.12	0.42	4.20	0.42	84.05	6.30	8.82	0.00

	**Tactile**							
	**Happiness**				**Sadness**			
	**Correct**	**Sadness**	**Fear/Threat**	**Peacefulness**	**Correct**	**Happiness**	**Fear/Threat**	**Peacefulness**

Controls	55.87	8.82	26.07	8.82	33.18	21.43	25.63	18.49
Musicians	54.63	6.30	19.71	16.81	23.51	15.97	25.21	31.93

	**Fear/Threat**				**Peacefulness**			
	**Correct**	**Happiness**	**Sadness**	**Peacefulness**	**Correct**	**Happiness**	**Sadness**	**Fear/Threat**

Controls	45.78	21.85	24.79	13.39	13.85	41.60	3.00	23.11
Musicians	55.03	9.24	19.33	13.03	35.71	27.31	16.39	17.65

*Bold and ^∗^*p* < 0.05.*

## Discussion

The main objective of the present study was to investigate auditory, tactile and auditory-tactile identification of emotion in musicians versus non-musicians. A significant difference between groups was found, with musicians showing better emotion identification for fear/threat in the auditory condition and for peacefulness in both the auditory and tactile conditions. Additionally, even if the difference does not remain significant after correcting for multiple comparisons, the trend indicates a possible gain from adding tactile stimulation to the auditory stimuli in peacefulness condition for controls (12%), but not for musicians (under 0%).

The significant differences found between controls and musicians can be linked to the complexity of the emotions displayed. It is well-known that happiness and sadness are the easiest emotions to identify because they are mainly based on tempo (see Terwogt and Van Grinsven, 1991). As such it is not surprising that results revealed no difference between controls and musicians for happy (auditory, auditory-tactile and tactile) and sad (auditory and auditory-tactile) conditions as there were ceiling effects. The average performance for sad for tactile stimulation did not differ between groups, but also, did not differ from chance for both groups. A more sensitive task would be needed to determine whether musical expertise can lead to more accurate identification of these emotions via auditory, tactile and auditory-tactile stimulation. Fear/threat is a musically less straightforward emotion than happiness and sadness (Vieillard et al., 2008; Tan et al., 2017). Hence, compared to controls, musicians more accurately identified that emotion in the auditory condition. In the same vein, the most complex and ambiguous emotion displayed in the sample melodies, namely peacefulness (Vieillard et al., 2008; Tan et al., 2017), was more accurately identify by musicians than by controls in both the auditory and the tactile conditions.

Results from the auditory condition are consistent with the extensive literature demonstrating that musical training leads to brain plasticity and can improve music processing as measured by pitch, timing and timbre discriminations (for a review see Kraus and Chandrasekaran, 2010). Since the identification of emotions in music is based on psychoacoustic cues and musical features, the enhanced performance of musicians in the auditory condition was also to be expected. Furthermore, an important component of musical training is aimed at understanding and experiencing the full range of emotional meaning and expressiveness, however faint, of a musical performance (Castro and Lima, 2014). As such, it is not surprising that improved performance was only found in conditions where musicians had to identify subtle emotional qualities.

One recent study have investigated recognition of emotions in an auditory-only stimulation condition. They suggest a correlation between years of musical training and accuracy at identifying emotion in music and revealed a significant difference between groups for older musicians with respect to sad and fear emotions (Castro and Lima, 2014). It should be noted that a major limitation of this study was that the range of musical expertise of participants as measured in years was large (8–18 years), and that the average age of training onset was over 7 years of age, the known threshold beyond which music-induced structural changes and learning effects become less pronounced (for a review see Habib and Besson, 2009). As such the lesser musical expertise of their younger participants may explain why they could not find any significant differences between groups. In contrast, results from the present study were obtained with participants whose average age of learning onset was 7 years of age, and whose average number of years of active practice of music was 20.2 years. All participants were working or studying full-time in the field of music and can be considered professional musicians. In addition, the average age of the participants was 34.4 years for controls and 28.9 years for musicians, which corresponds to the younger group of Castro and Lima (2014).

The present study was the first to investigate the tactile identification of emotions in music. Results revealed that both musicians and controls were able to identify emotions via tactile stimulation only, which is in itself a new and major finding. No study to date has investigated purely tactile identification of emotion in music. The only existing study along these lines was performed by Branje et al. (2013) and suggests that multisensory stimulation can increase emotion perception in film. By using the Emoti-Chair, a device that induces vibration in the back of normal-hearing participants, they found increases in skin conductance levels when vibrotactile stimuli were added to audio/visual film content. They also observed that not only the intensity of vibration but also the frequency of the vibrotacile stimuli was playing a role in the observed reactions. The present study results are consistent with Branje et al. (2013) and further support the hypothesis that both controls and musicians are able to extract meaningful information from the frequency characteristics of a signal presented through vibrations only. Furthermore, for the emotion of peacefulness, results revealed a significant difference between musicians and controls for tactile stimulation. These results are consistent with a previous study from our laboratory, the first to demonstrate that musicians were better at discriminating frequencies via tactile stimulation applied to the hand (Sharp et al., 2019). The enhanced ability to identify peaceful emotions in music via tactile stimulation suggests that more complex processes are improved following long-term musical training. This hypothesis should be verified using other types of complex emotions that are easier to identify via tactile stimulation than peacefulness. Indeed, results in the peacefulness condition are above chance for musicians, but not for controls and the comparison of performance would be easier to interpret if both groups were above chance.

It is well-known that the frequency spectrum treated is more limited than that of the hair cells of the cochlea (1 to 1000 kHz) (Rovan and Hayward, 2000). Which musical components is perceived though the tactile modality remains a question of debate. Some studies suggest that non-musicians can detect different musical notes via the tactile modality (Hopkins et al., 2016) and that they can discriminate timbre (Russo et al., 2012). Furthermore, low frequencies in music are important to understanding beat and can be transmitted via vibrotactile devices (Van Dyck et al., 2013; Tranchant et al., 2017). All these psychoacoustic cues are known to be transmitted via the tactile modality and are all important for emotion identification in music. Further study should investigate if other cues are used in the identification of emotion in the tactile domain or if some of these cues are more important than the others. All these studies support our results suggesting that non-musicians and musicians are able to identify emotion via tactile stimulation only.

Finally, the lack of significant difference between musicians and non-musicians in the auditory-tactile condition can be explained by the trend for controls toward exhibiting gain from tactile stimulation compared to musicians, as the latter were already too skilled in the auditory domain to benefit from tactile stimulation. Further studies should use more complex emotional stimuli to assess whether there could be a tactile gain for musicians, and investigate whether non-musicians’ performance could become similar to that of musicians with training and feedback.

## Data Availability

The raw data supporting the conclusions of this manuscript will be made available by the authors, without undue reservation, to any qualified researcher.

## Ethics Statement

This study was carried out in accordance with the recommendations of Research Committee for Health Sciences of the University of Montreal and the Center for Interdisciplinary Research in Rehabilitation of Greater Montreal with written informed consent from all subjects. All subjects gave written informed consent in accordance with the Declaration of Helsinki. The protocol was approved by the Research Committee for Health Sciences of the University of Montreal and the Center for Interdisciplinary Research in Rehabilitation of Greater Montreal.

## Author Contributions

AS and FC designed and performed the experiment. All authors wrote the manuscript, discussed the results and implications, and commented on the manuscript at all stages.

## Conflict of Interest Statement

The authors declare that the research was conducted in the absence of any commercial or financial relationships that could be construed as a potential conflict of interest.
